# Editorial: Family men: fathers as coparents in diverse contexts and family structures, volume II

**DOI:** 10.3389/fpsyg.2024.1504391

**Published:** 2024-11-26

**Authors:** Sarah E. DeMartini, Martin I. Gallegos, Lauren Altenburger, Nicola Carone

**Affiliations:** ^1^Department of Psychology, California State University, Chico, CA, United States; ^2^University of Texas at San Antonio, San Antonio, TX, United States; ^3^Department of Human Development and Family Studies, Penn State Shenango, Sharon, PA, United States; ^4^Department of Systems Medicine, University of Rome Tor Vergata, Rome, Italy

**Keywords:** fathers, coparenting, partner dynamics, masculinity, trauma informed approaches, motherhood

Fathers' involvement in childrearing is on the rise despite roadblocks set by gender norms, institutions, policies, and partner dynamics (Volling and Palkovitz, 2021). Studying coparenting offers insights into parents' joint efforts in caregiving responsibilities (McHale and Jones, 2021). The second volume for our Research Topic welcomed articles that considered the challenges fathers face in relation to coparenting, such as partner dynamics; child and adolescent outcomes; diverse family structures (e.g., same- and different-sex couples, fathers of one or multiple children, divorced, or intact families); and varied racial, ethnic, and cultural backgrounds. Ten articles with a global reach are highlighted that explore modern fatherhood via rigorous methodologies.

Seven articles in our Research Topic were quantitative research studies that explored the impact of fathers from diverse family contexts and dynamics.

Parental mental health, reflective functioning, and coparenting during the transition to parenthood in relation to children's socioemotional development were cross-sectionally examined in an Australian sample (De Palma et al.). An indirect effect of general reflective functioning (certainty) on child socioemotional development via parental reflective functioning (pre-mentalizing) emerged. Also, an indirect effect of negative coparenting on child socioemotional development via parental reflective functioning (pre-mentalizing) was found.

Ji et al. aimed to identify the mechanisms that impact maternal positive coparenting on adolescents' ego-identity in a Chinese sample. Structural equation modeling revealed that peer relationships mediated the relationship between maternal positive coparenting and adolescent ego-identity. Fathers' marital satisfaction and peer relationships also chain-mediated the role between maternal positive coparenting and adolescent ego-identity.

Scheifele et al. explored how fatherhood and masculinity beliefs, social support, and environmental factors influenced men's formation of parental leave intentions across the transition to parenthood in Belgium and Germany. Hierarchical regression models suggested that men who felt more support from their partners to utilize parental leave options had increased desire and intention to use their parental leave, as well as longer planned length of leave.

McHale et al. conducted a study in the United States in which they identified a need for more trauma-informed support for families. They outlined the planning process, Trauma-Informed Family Centered principles training series, and profile assessments they underwent with local organizations to achieve this aim. Through direct collaboration with several organizations, they were able to coordinate, train, consult, self-monitor, and problem solve to effectively deliver Trauma-Informed services.

McHale et al. proposed a novel approach and rating system to aid practitioners and supervisors in assessing the quality of coparenting in couples group interventions in a United States sample in which couples were English- or Spanish-speaking. Results indicated that, over time, both English- and Spanish-speaking couples discussed coparenting related challenges; process-oriented responses were especially helpful in these circumstances.

Puglisi et al. made use of physiological assessments to investigate the association between parent-child interactive synchrony and infants' vagal tone in a Switzerland-based sample. Structural equation models suggested that variations in parent-child synchrony were related to variations in infants' vagal tone during mother-child interactions; this finding was only consistently found when mothers and their infants interacted after fathers did with their children.

The impact of parental gender and caregiving roles on positive and negative affect during interactions with their infant for same- and different-sex couples were investigated by Leter et al.. It was further investigated whether parenting stress, infant temperament, having a singleton vs. twin, and country of residence (Netherlands, France, or the United Kingdom) were associated with parental positive and negative affect. Mixed linear models revealed country of origin to be the sole predictor of parental negative and positive affect.

Our Research Topic has one Brief Research Report, which highlights ethical, original research in a succinct manner.

Kuo et al. used actor-partner interdependence moderation models to examine the role of caregiving to explain the relation between parents' marital satisfaction and coparenting quality in a United States sample. Both parents' caregiving identities interacted with their own reports of marital satisfaction to predict mothers' (but not fathers') perceptions of coparenting quality. Interestingly, both parents' caregiving identity only related to their partner's perceptions of coparenting quality but not their own perceptions.

Our Research Topic contains one Policy and Practice Review that highlights the importance of including both parents' reports in analyses.

Sandberg outlines the positive trajectory of father involvement in Denmark across recent decades. Despite this increase and the benefits of father involvement for child adjustment, Sandberg points out that father involvement and shared parenting are relatively low following divorce in Denmark. To understand factors that contribute to this phenomenon, several Danish guidelines/practices that may hinder father involvement and shared parenting in post-divorce families were examined.

The last article submission type for our Research Topic is a Community Case Study, where intersectional practices are discussed in relation to improving the health and wellbeing of a population.

Hudson and Brotherson identify systemic adversities and historical trauma amongst fathers in Native American and Afro-Caribbean communities and their ability to fulfill coparenting roles. The aim of this case study was to suggest a cross-cultural adaptation of the Fatherhood is Sacred Program, originally developed for Native American families, to Afro-Caribbean families.

The second volume of our Research Topic emphasizes the gendered effects on coparenting efforts, with contextual emphasis on the push and pull of societal pressures, cultural discrepancies, and the influence of one's partner on fathering efforts. The clinical, educational, and therapeutic recommendations from the ten articles advise on bridging the gap between applied fields and research sciences. In turn, we think it is important to embrace applied methodologies and acknowledge their contributions in and outside the format of Original Research to advance our understanding of fatherhood. It is important to recognize the shortcoming of our Research Topic, having only one study with a same-gender sample, as the heteronormativity of fatherhood lacks important perspectives in aiding fathers that represent broader society. It is our hope that readers will find our second research volume to be a springboard from which to advance further work on fathers as coparents in diverse contexts.
